# Altered Gut Microbiota Contributes to Acute-Respiratory-Distress-Syndrome-Related Depression through Microglial Neuroinflammation

**DOI:** 10.34133/research.0636

**Published:** 2025-03-19

**Authors:** Bowen Zhu, Zheng Gu, Hongbin Hu, Jie Huang, Zhenhua Zeng, Haoxuan Liang, Ziyi Yuan, Shiwei Huang, Yuetan Qiu, Xiang-Dong Sun, Youtan Liu

**Affiliations:** ^1^ Department of Anesthesiology, Shenzhen Hospital, Southern Medical University, Shenzhen, China.; ^2^ Key Laboratory of Mental Health of the Ministry of Education, Guangdong-Hong Kong-Macao Greater Bay Area Center for Brain Science and Brain-Inspired Intelligence, Guangdong-Hong Kong Joint Laboratory for Psychiatric Disorders, Guangdong Province Key Laboratory of Psychiatric Disorders, Guangdong Basic Research Center of Excellence for Integrated Traditional and Western Medicine for Qingzhi Diseases, Department of Neurobiology, School of Basic Medical Sciences, Southern Medical University, Guangzhou, China.; ^3^ Department of Critical Care Medicine, Nanfang Hospital, Southern Medical University, Guangzhou, China.

## Abstract

Acute respiratory distress syndrome (ARDS) survivors often suffer from long-term psychiatric disorders such as depression, but the underlying mechanisms remain unclear. Here, we found marked alterations in the composition of gut microbiota in both ARDS patients and mouse models. We investigated the role of one of the dramatically changed bacteria—*Akkermansia muciniphila* (*AKK*), whose abundance was negatively correlated with depression phenotypes in both ARDS patients and ARDS mouse models. Specifically, while fecal transplantation from ARDS patients into naive mice led to depressive-like behaviors, microglial activation, and intestinal barrier destruction, colonization of *AKK* or oral administration of its metabolite—propionic acid—alleviated these deficits in ARDS mice. Mechanistically, *AKK* and propionic acid decreased microglial activation and neuronal inflammation through inhibiting the Toll-like receptor 4/nuclear factor κB signaling pathway. Together, these results reveal a microbiota-dependent mechanism for ARDS-related depression and provide insight for developing a novel preventative strategy for ARDS-related psychiatric symptoms.

## Introduction

Acute respiratory distress syndrome (ARDS) is characterized by extensive pulmonary inflammation and edema, commonly leading to acute respiratory failure [1]. It affects approximately 10% of patients in intensive care units (ICUs) worldwide, with mortality rates reaching as high as 30% to 40% [2]. Notably, more than a third of patients develop psychiatric disorders, such as anxiety or depression, 6 months after discharge [3–6]. However, the pathological mechanisms underlying ARDS-related psychiatric symptoms remain unclear, and effective prevention and treatment strategies are still lacking [6–8].

It has been reported that the inflammatory response in ARDS can disrupt the blood–brain barrier, allowing harmful substances such as inflammatory cytokines to enter the brain, leading to neurological dysfunctions [9,10]. Growing evidence suggests that the gut microbiota, known as the “second brain”, regulates neuroinflammation through the microbiota–gut–brain axis, impacting the host’s immune system and behaviors [11–14]. For instance, *Akkermansia muciniphila* (*AKK*), a recognized probiotic within the gut microbiota, has been shown to restore gut microbiota balance, rebuild the integrity of the intestinal mucosal barrier, and regulate host immunity and neuroinflammation through short-chain fatty acids (such as acetic acid and propionic acid [PA]) [15], therefore alleviating neural damage and behavioral abnormalities in various disorders, including epilepsy, amyotrophic lateral sclerosis, and autism [16–19]. Notably, recent studies suggest that ARDS patients exhibit gut microbiota dysbiosis [20,21]. Nevertheless, whether alterations in gut microbiota are involved in ARDS-related psychiatric symptoms awaits to be determined.

In the present study, we sought to explore the role of microbiota in the pathogenesis of ARDS-related psychiatric symptoms. By using 16S ribosomal DNA (rDNA) sequencing and untargeted metabolomics, we found dramatic alterations in the composition of gut microbiota and serum metabolites in both ARDS patients and mouse models, identifying that *AKK* and the metabolite PA were vital for ARDS-related depressive-like behaviors. Lastly, we investigated the underlying mechanisms by which PA regulates inflammation. Together, these results demonstrate an important role of gut microbiota, especially *AKK*, in ARDS-related emotional dysfunctions and shield light on developing new interventions.

## Results

### Gut microbiota and metabolites in feces and serum are altered in ARDS patients

**Fig. 1.**
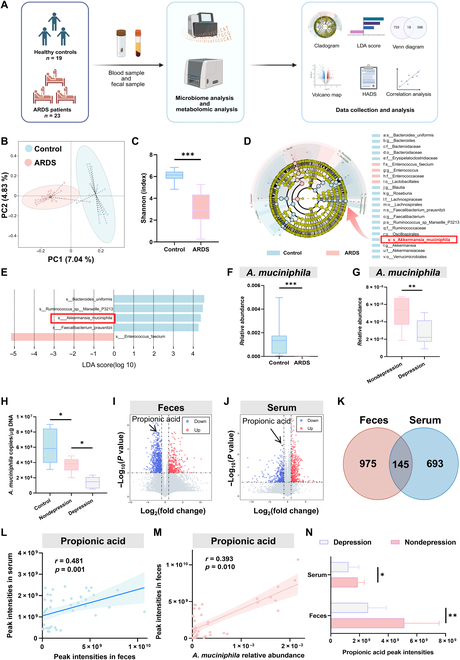
*Akkermansia muciniphila* (*AKK*) and propionic acid (PA) are down-regulated in acute respiratory distress syndrome (ARDS) patients with depression. (A) Schematic process of experimental design. Blood and stool samples were collected from 23 ARDS patients and 19 healthy volunteers. Samples were used for subsequent experiments. (B) β diversity of principal component analysis (PCA). (C) Analysis of the α diversity of gut microbiota in the feces of the 2 groups. (D and E) Linear discriminant analysis effect size (LEfSe) was used to find species with significant differences at all taxonomic levels of the gut microbiota in the feces of 2 groups. The comparison strategy was one-against-all, and the linear discriminant analysis (LDA) threshold was 2. (F) The relative abundance of *AKK* in the control group and ARDS group. (G) The relative abundance of gut microbiota in the ARDS depression group and ARDS nondepression group. (H) The concentration of *AKK* in feces was assessed by real-time polymerase chain reaction (qPCR) (*n* = 6). (I and J) The differential metabolites in serum and feces of ARDS and healthy control were revealed by volcano plots. *X* axis: log_2_ fold change (log_2_ FC); *Y* axis: −log_10_(adjusted *P* value). The cutoff was set using an FC of 2 and an adjusted *P* value of 0.01. (K) The Venn diagram indicates the intersection of 2 groups of changed metabolites. (L) Spearman’s correlation of PA in serum and feces. (M) Spearman’s correlation between *AKK* and PA in feces. (N) Relative levels of PA in serum and feces in depressed and nondepressed groups (Student *t* test, *P < 0.05, **P < 0.01, and ***P < 0.005; ns means not significant). HADS, Hospital Anxiety and Depression Scale.

To determine whether the microbiota plays a role in ARDS-related psychiatric symptoms, we first assessed the gut microbiota profile and changes in fecal and serum metabolites from 19 healthy volunteers (control) and 23 ARDS patients (Fig. 1A). Healthy control groups and ARDS groups did not differ significantly in gender or age (Table S1). Of note, the procalcitonin and C-reactive protein levels of ARDS patients were dramatically elevated, indicating a high inflammatory response (Table S1). Principal component analysis indicated that the composition of the gut microbiota was different between control and ARDS patients (Fig. 1B). In addition, the Shannon index was lower in ARDS patients than in healthy controls (Fig. 1C), suggesting a decreased alpha diversity of microbiota. Importantly, linear discriminant analysis (LDA) and LDA effect size analysis showed that the relative abundance of *Bacteroides uniformis*, *AKK*, and *Faecalibacterium prausnitzii* was decreased, while that of *Enterococcus faecium* was increased in ARDS patients (Fig. 1D and E). Notably, 10 of 23 ARDS patients exhibited depression symptoms 3 months after discharge (Table S1). The relative abundance of *AKK* in ARDS patients with depression symptoms was significantly decreased compared with that in patients without depression symptoms, while there was no difference in *B. uniformis*, *P. prausnitzii*, or *E. faecium* (Fig. 1F and G and Fig. S1A). The changes in *AKK* were verified by real-time polymerase chain reaction (qPCR) analysis (Fig. 1H). These results suggest an involvement of *AKK* in the development of ARDS-related depression symptoms.

Previous studies indicated the important roles of metabolites produced by gut microbiota for regulating brain functions [22]. To determine whether metabolites are altered in ARDS, we performed untargeted metabolomic analysis of serum and fecal samples; 1,120 and 838 metabolites in total were changed in feces and serum of ARDS patients, respectively, compared with healthy controls (Fig. 1I to K). Among these, 18 and 21 metabolites were down-regulated and up-regulated in both fecal and serum samples, respectively (Fig. S1B and C and Table S2). Of note is that the level of PA, which is a major metabolite of *AKK* and has been shown to exhibit neuroprotective effects [23,24], was significantly lower in both the serum and feces of ARDS patients (Fig. 1I and J). The level of PA in feces was positively correlated with that in serum and the relative abundance of *AKK* (Fig. 1L and M and Fig. S1D). More importantly, we found that the PA levels in the feces and serum were significantly decreased in the depressed group compared to those in the nondepressed group (Fig. 1N). Together, these results suggest potential associations among decreased relative abundance of *AKK* and the level of its metabolite PA and ARDS-related depression symptoms.

### The ARDS mouse model exhibits depressive-like behaviors and microglial activation in the brain

**Fig. 2.**
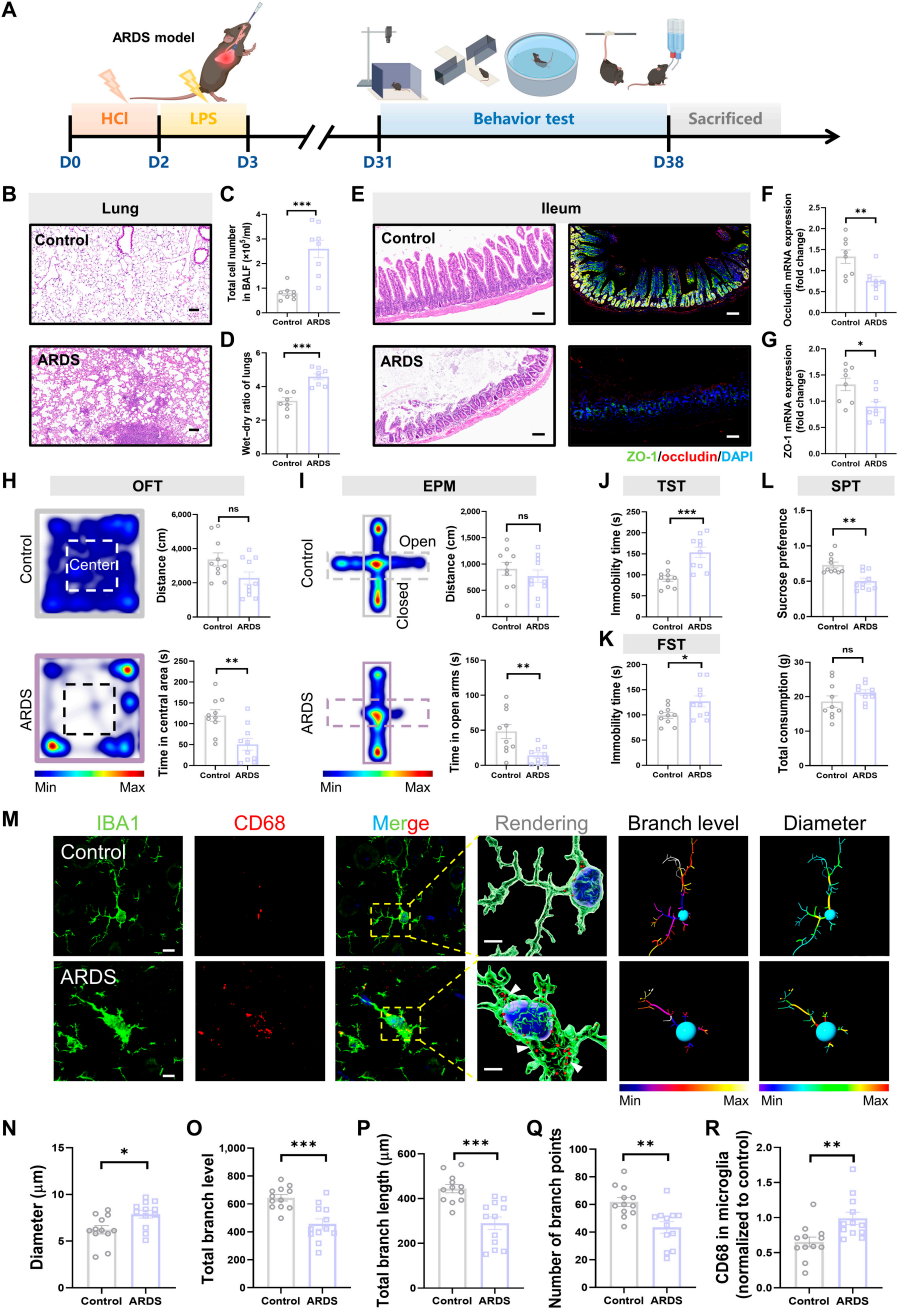
Long-term depressive-like behavior disorder with breakdown of gut barrier integrity and microglial activation in ARDS mice. (A) The flowchart of the experiment shows the “2-hit” ARDS mouse model and the detection time of each cognitive behavior. (B) Hematoxylin–eosin staining was used to display the severity of lung injury. Scale bar = 100 μm. (C) and (D) are correlated with (B); the total cell number in bronchoalveolar lavage fluid (BALF) and the wet–dry ratio of lungs were calculated. (E) Immunofluorescence staining was used to detect the expression level of the tight junction protein zona occludens 1 (ZO-1, green) and occludin (red) in the mouse ileal tissues in each group. Scale bar = 50 μm. (F and G) Correlation with (E) and the messenger RNA (mRNA) expression levels of ZO-1 and occludin in the mouse ileal tissues were calculated. (H) Heatmap of mouse behavior in the open field test (OFT). Summarized data of distance and time in center area from the indicated group. (I) Heatmap of mouse behavior in the elevated plus maze (EPM). Summarized data of distance and time in open arm from the indicated group. (J) Measurement of immobility time in the tail suspension test (TST). (K) Measurement of immobility time in the forced swimming test (FST). (L) Summarized data of sucrose preference and total consumption from the indicated group. (M) Immunofluorescence micrographs of prefrontal cortex microglia (Iba-1, green; CD68, red). Inside the yellow box is a typical microglia. Scale bar = 10 μm. In the rendering, the white arrows indicate CD68. Scale bar = 5 μm. (N to R) Quantification of the branch diameter, branch level, branch length, number of branch points, and CD68 of prefrontal cortex microglia (both groups, *n* = 12 sections from 5 mice) (Student *t* test, *P < 0.05, **P < 0.01, and ***P < 0.005; ns means not significant). LPS, lipopolysaccharide; DAPI, 4′,6-diamidino-2-phenylindole; SPT, sucrose preference test; Iba-1, ionized calcium binding adaptor molecule 1.

To investigate the underlying mechanisms for ARDS-related depression, we developed a “2-hit” mouse model to mimic the pathophysiological process observed in clinical ARDS patients [25] (Fig. 2A). We found that the survival rate of ARDS mice was stabilized at a level of 60.1% 40 d after LPS injection (Fig. S2A). The total cell number in bronchoalveolar lavage fluid and the wet-to-dry weight ratio of lungs were increased, and SpO_2_ levels were significantly decreased in ARDS mice, indicating persistent lung injury (Fig. 2B to D and Fig. S2B). Meanwhile, the immunofluorescent assay confirmed a significant reduction in ileal tight junction proteins zona occludens 1 (ZO-1) and occludin in ARDS mice (Fig. 2E to G), suggesting disrupted intestinal integrity. Behaviorally, the time that mice spent in the central area in the open field test and that in the open arm in the elevated plus maze (EPM) were both reduced (Fig. 2H and I). Moreover, the immobility time of ARDS mice in the forced swimming test (FST) and tail suspension test was significantly increased compared with that of controls (Fig. 2J and K). ARDS mice also exhibited reduced sucrose preference, whereas there was no significant difference in total consumption (Fig. 2L). These results indicate that ARDS mice exhibit depressive-like behaviors. It is known that microglia, the innate immune cells in the brain, play a key role in regulating depressive-like behaviors in mice [26]. We found that the microglia in the prefrontal cortex of ARDS mice were in an activated state, characterized by a decrease in branch levels, branch length, and soma diameter but an increase in CD68-positive fluorescence intensity (Fig. 2M to R). Together, these findings suggest that ARDS mice exhibit microglial activation in the brain and depressive-like behaviors.

### The relative abundance of *AKK* and the PA level were associated with depressive-like behaviors in ARDS mice

**Fig. 3.**
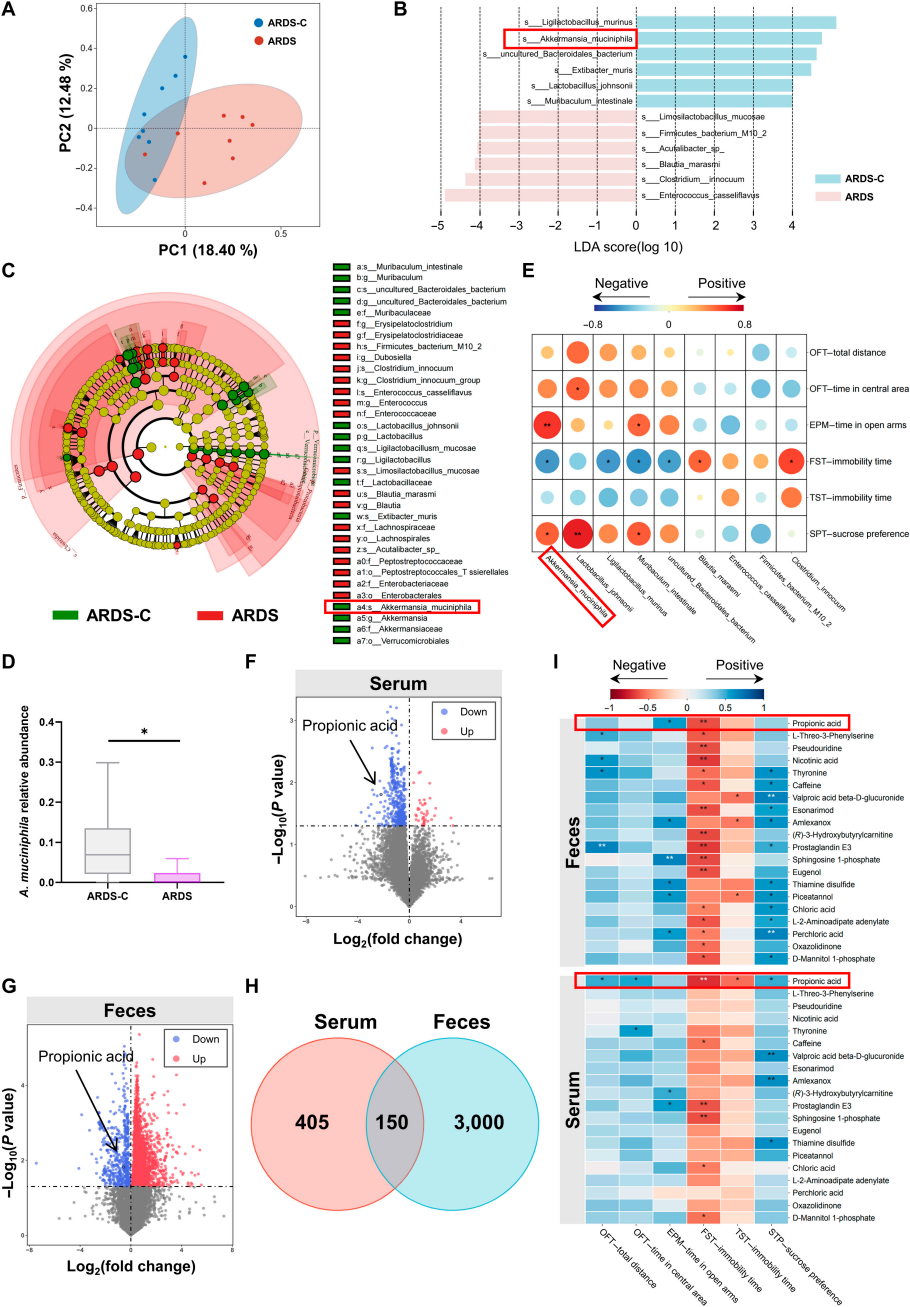
*AKK* and metabolite PA are decreased in ARDS mice, which are associated with depressive behaviors. (A) β diversity of PCA. PC1 and PC2 can separate control mice from ARDS mice. (B and C) LEfSe was used to find species with significant differences at all taxonomic levels of the gut microbiota in the feces of the 2 groups of mice. The comparison strategy was one-against-all, and the LDA threshold was 2. (D) The relative abundance of *AKK* in the ARDS group and control group. (E) Correlation analysis heatmap of gut microbiota and measures of depression indicators in mice. Red represents a positive correlation; blue represents a negative correlation. (F and G) The differential metabolites in the serum and feces of ARDS and healthy control were revealed by volcano plots. *X* axis: log_2_ fold of change (log_2_ FC); *Y* axis: −log_10_(adjusted *P* value). The cutoff was set using an FC of 1 and an adjusted *P* value of 0.05. (H) The Venn diagram indicates the intersection of the 2 groups of changed metabolites. (I) Correlation analysis heatmap of fecal and serum metabolites in mice with depression indicators. Blue represents a positive correlation; red represents a negative correlation (Student *t* test, *P < 0.05 and **P < 0.01; ns means not significant). ARDS-C, control group.

Using 16S rDNA sequencing, we found that the microbiota composition in the ARDS mice was different from that in control mice (Fig. 3A). In line with the results from human patients, *AKK* was also down-regulated in the ARDS group and positively correlated with time in the open arm in the EPM and sucrose preference in the sucrose preference test but negatively correlated with immobility time in the FST (Fig. 3B to E). We further performed untargeted metabolomic analysis on serum and fecal samples of mice. Compared with the control group, the ARDS group showed 555 and 3,150 metabolites changed in serum and feces (Fig. 3F to H). Specifically, 40 serum metabolites and 2,531 fecal metabolites were up-regulated, while 515 serum metabolites and 619 fecal metabolites were down-regulated in the ARDS group (Fig. 3F and G). Notably, PA was significantly down-regulated in both the serum and feces of ARDS mice (Fig. 3F and G), which is consistent with the findings observed in human samples (Fig. S3A and B). Interestingly, PA was correlated with depressive-like behaviors (Fig. 3I). PA is one of the typical short-chain fatty acids and is metabolized by many microorganisms [27]. We measured the supernatant of *AKK* cultures and found that PA levels were significantly elevated in the *AKK* supernatant compared to those in the control group (Fig. S4). This result indicates that *AKK* is capable of producing PA, in line with previous reports [24,28]. Collectively, these results suggest an involvement of *AKK* and PA in ARDS-related depressive-like behaviors.

### Fecal microbiome transplant from ARDS patients is sufficient to induce depressive-like behavior in naive mice

**Fig. 4.**
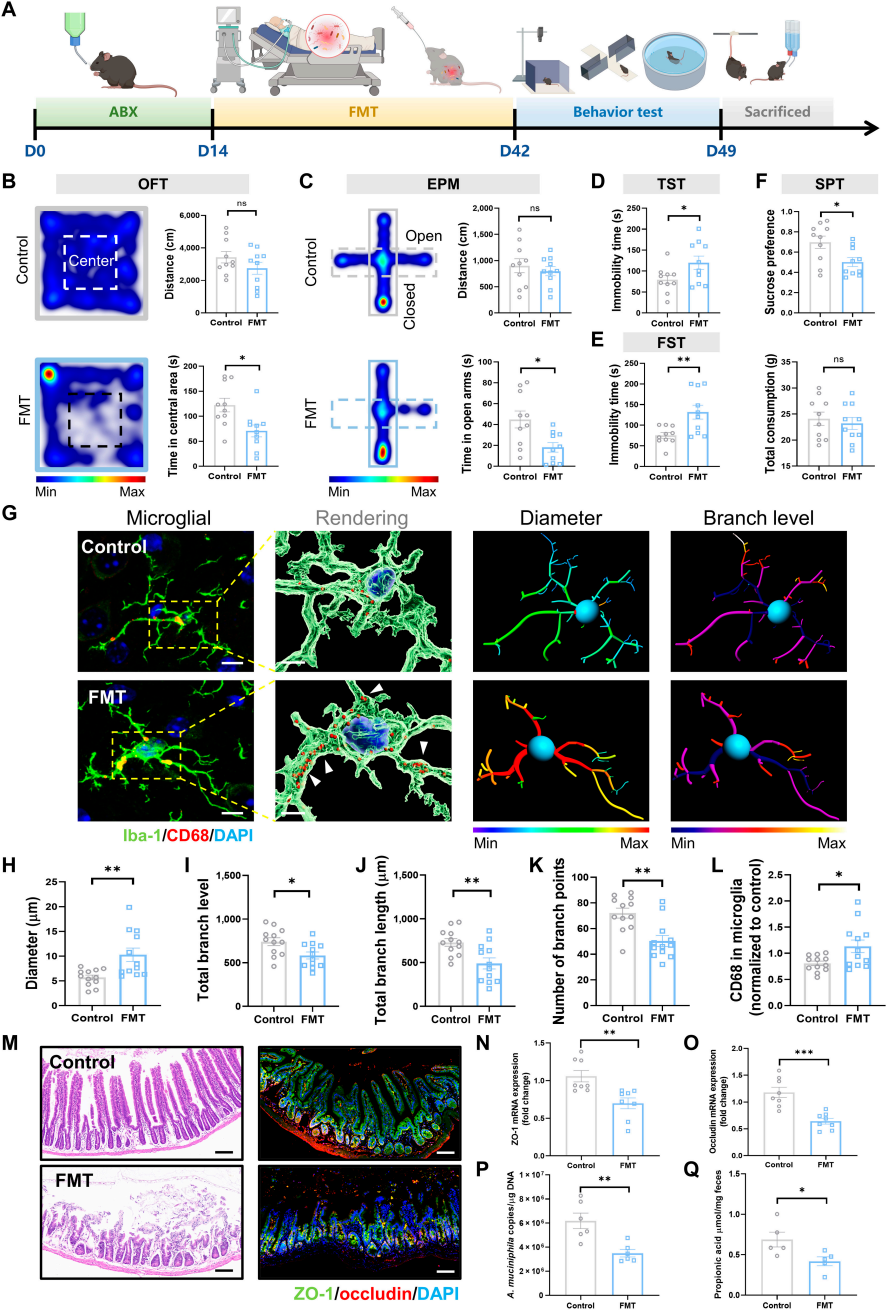
Transplantation of the gut microbiota from ARDS patients to healthy mice leads to microglial activation, intestinal barrier damage, and depression-like behaviors. (A) Schematic and timeline of fecal microbiota transplant (FMT) and behavior tests. (B) Heatmap of mouse behavior in OFT. Summarized data of distance and time in center area from the indicated group. (C) Heatmap of mouse behavior in the EPM. Summarized data of distance and time in the open arm from the indicated group. (D) Measurement of immobility time in TST. (E) Measurement of immobility time in FST. (F) Summarized data of sucrose preference and total consumption from the indicated group. (G) Immunofluorescence micrographs of prefrontal cortex microglia (Iba-1, green; CD68, red). Inside the yellow box is a typical microglia. Scale bar = 10 μm. In the rendering, the white arrows indicate CD68. Scale bar = 5 μm. (H to L) Quantification of the diameter, branch level, branch length, number of branch points, and CD68 of prefrontal cortex microglia (both groups, *n* = 12 sections from 5 mice). (M) Immunofluorescence staining was used to detect the expression level of the tight junction protein ZO-1 (green) and occludin (red) in the mouse ileal tissues in each group. Scale bar = 50 μm. (N and O) Correlation with (M) and the mRNA expression levels of ZO-1 and occludin in the mouse ileal tissues were calculated. (P) The concentration of *AKK* in cecal content was assessed by qPCR. (Q) The contents of PA in the feces (Student *t* test, *P < 0.05 and **P < 0.01; ns means not significant). ABX, antibiotics.

To investigate whether alterations in the gut microbiota are responsible for the depressive-like behaviors in the ARDS mice, we colonized C57 naive mice with fecal microbiome from ARDS patients with depression symptoms or healthy control subjects. Behavioral tests were performed 4 weeks after fecal microbiome transplant (FMT) (Fig. 4A). The open field test and EPM results showed that FMT mice spent less time in the center area and open arms compared with the control group (Fig. 4B and C). In the tail suspension test and FST, the immobility time of FMT mice was increased (Fig. 4D and E). In addition, the FMT mice exhibited a decreased sucrose preference, whereas there was no significant difference in total consumption (Fig. 4F). These behavioral results indicate that ARDS microbiota is sufficient to cause depressive-like behaviors in naive mice. We also found activated microglia in the prefrontal cortex, as the branch levels, branch length, and soma diameter of microglia in FMT mice were reduced, whereas the proportion of CD68-positive cells was increased (Fig. 4G to L). Moreover, the levels of inflammatory factors, including interleukin 6 (IL-6), interleukin 1 beta (IL-1β), lipopolysaccharide (LPS), and tumor necrosis factor alpha (TNF-α), were increased in the FMT group (Fig. S5A to D). Furthermore, the expressions of ZO-1 and occludin were significantly decreased in the FMT mice compared with those in control mice (Fig. 4M to O). In consistency with this, the fecal *AKK* and PA were also decreased in the FMT mice.

### *AKK* colonization reverses the reduction of PA, microglia activation, and depressive-like behaviors

**Fig. 5.**
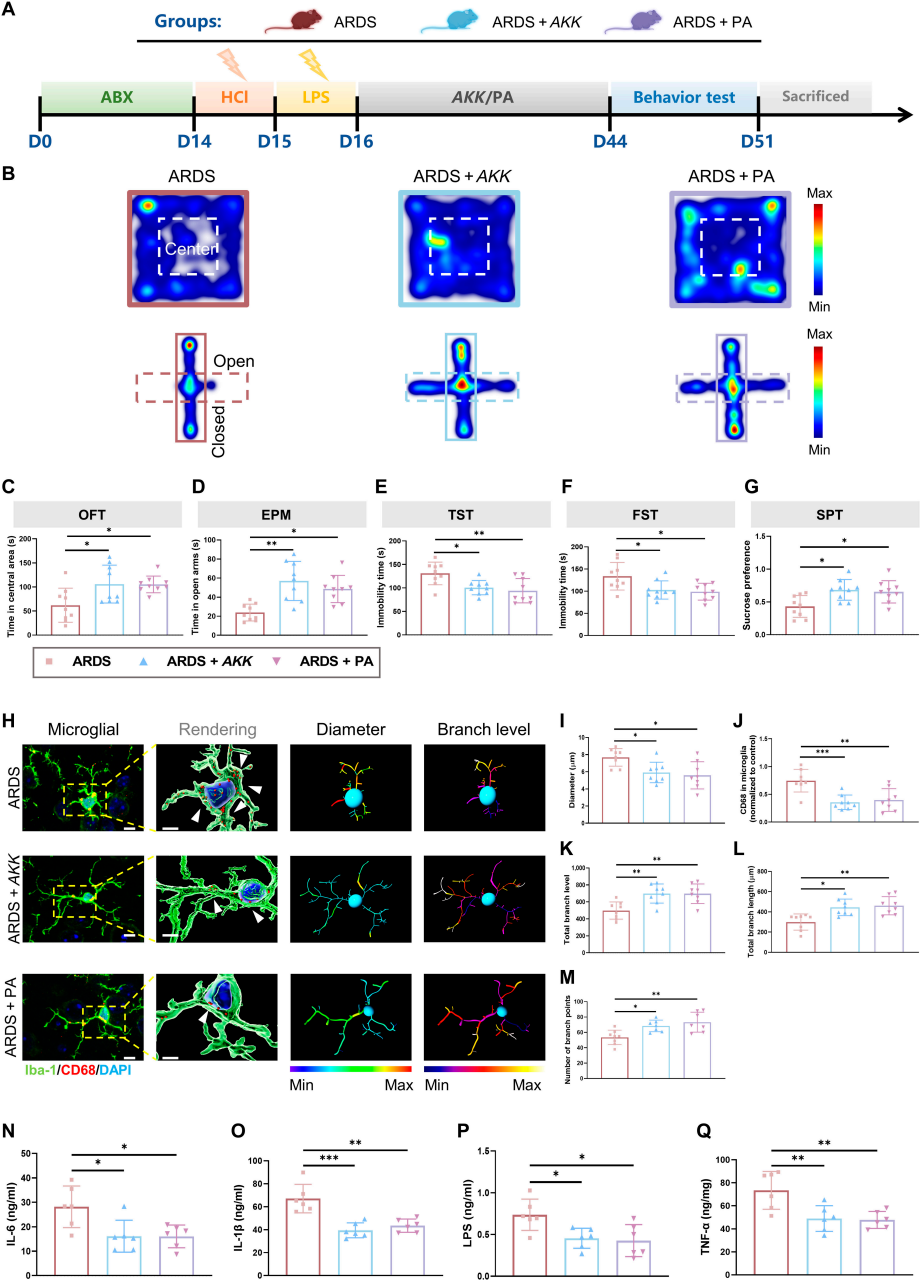
*AKK* or PA treatment reduces the inflammation response and restores the behavior disorder of ARDS mice. (A) The flowchart shows the time points at which the mice were treated with *AKK* or PA after “2-hit” modeling, behavioral testing, and specimen collection (all groups, *n* = 9). (B) Representative heatmap of mouse behavior in the OFT and EPM. (C to G) Summarized data of mouse behavior in OFT, EPM, TST, FST, and SPT. (H) Immunofluorescence micrographs of prefrontal cortex microglia (Iba-1, green; CD68, red). Inside the yellow box is a typical microglia. Scale bar = 10 μm. In the rendering, the white arrows indicate CD68. Scale bar = 5 μm. (I to M) Quantification of the diameter, branch level, branch length, number of branch points, and CD68 of prefrontal cortex microglia (all groups, *n* = 8 sections from 5 mice). (N to Q) The levels of interleukin 6 (IL-6), interleukin 1 beta (IL-1β), LPS, and tumor necrosis factor alpha (TNF-α) in the cerebral cortex (*n* = 6). (one-way analysis of variance with Tukey’s multiple-comparisons tests, *P < 0.05, **P < 0.01, and ***P < 0.005; ns means not significant).

**Fig. 6.**
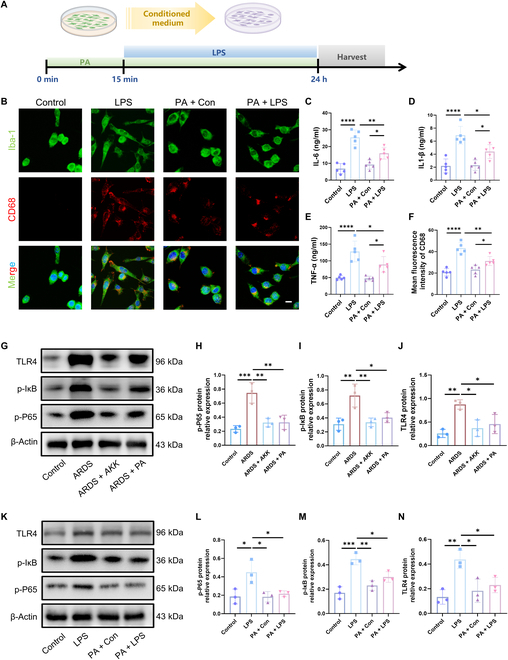
*AKK* and PA improve neuroinflammation by inhibiting Toll-like receptor 4 (TLR4)/nuclear factor κB (NF-κB) signaling in microglia. (A) PA treatment of primary microglia. (B) Immunofluorescence micrographs of microglia (Iba-1, green; CD68, red). Scale bar = 15 μm. (C to F) Levels of IL-6, IL-1β, and TNF-α and fluorescence intensity of CD68 in the microglia (*n* = 5). (G to J) Relative protein levels of TLR4, phospho-IκB (p-IκB), and p-P65 in mouse prefrontal cortex tissue by western blot (*n* = 3). (K to N) Relative protein levels of TLR4, p-IκB, and p-P65 in microglia by western blot (*n* = 3) (Student *t* test, *P < 0.05, **P < 0.01, ***P < 0.005, and ****P < 0.001; ns means not significant).

To determine whether application of *AKK* and PA could ameliorate microglia activation and depressive-like behaviors in ARDS mice, we colonized ARDS mice pretreated with antibiotics with *AKK* or inject ARDS mice with PA (Fig. 5A). Four weeks later, we found that the application of *AKK* and PA increased the levels of PA in the serum of mice (Fig. S6A). Importantly, the depressive-like behaviors in ARDS mice were alleviated by *AKK* or PA supplementation (Fig. 5B to G and Fig. S6E to G). Furthermore, the activation state of microglia in ARDS mice was restored, as evidenced by decreased soma diameter and proportion of CD68-positive cells, elongated branches, and increased branch levels (Fig. 5H to M). We also found that the cytokines, including IL-6, IL-1β, LPS, and TNF-α, in the prefrontal cortex were decreased (Fig. 5N to Q). Histologically, the disrupted colonic structure and lung injury were alleviated in the ARDS + *AKK* group and ARDS + PA group (Fig. 6B and C). The ZO-1 and occludin expressions were also increased (Fig. S6D). Collectively, these results suggest that *AKK* treatment restored PA levels and microglia activation, which eventually leads to the amelioration of depressive-like behaviors in ARDS mice.

### PA and *AKK* mitigate microglial activation via TLR4/NF-κB pathway inhibition

To further explore the underlying mechanism of the beneficial effects of PA on microglia activation, we used LPS to activate microglia in culture with supplementation of PA (Fig. 6A). The microglia in the LPS group were characterized by thick and short cell bodies, indicative of an acquired pro-inflammatory M1 amoeboid morphology (Fig. 6B). After treatment with PA, LPS-induced morphological changes in microglia were attenuated (Fig. 6B). Moreover, LPS exposure of microglia led to increased secretion of IL-6, IL-1β, and TNF-α and fluorescence intensity of CD68. However, inflammatory factor levels in microglia were decreased by PA (Fig. 6C to F). It has been reported that Toll-like receptor 4 (TLR4) is a type I transmembrane protein expressed on microglia that recognizes and responds to LPSs [29]. It could initiate intracellular signaling through the nuclear factor κB (NF-κB) pathway, leading to the release of inflammatory factors [30]. To examine the potential involvement of the TLR4/NF-κB pathway in the pathogenesis of ARDS-related depressive-like behaviors and whether it could be affected by *AKK* and PA treatment, we assessed the levels of TLR4, p-IκB, and p-P65 proteins with western blotting analysis. The results showed that their levels were increased in the prefrontal cortex of ARDS mice and LPS-treated microglia, which were significantly ameliorated by the treatment with *AKK* and PA (Fig. 6G to N). Note that the inhibitory effect of PA on microglia activation as well as the expressions of TLR4, p-IκB, and p-P65 were blocked by TAK-243, an antagonist of the TLR4 receptor (Fig. S7). These results indicate that PA alleviates microglia activation by inhibiting the TLR4/NF-κB inflammatory pathway, which may contribute to the protective effects of PA on ARDS-related depression. To evaluate the potential interaction between PA and TLR4, we performed molecular docking analysis. The results showed that PA and TLR4 exhibited a great binding power, with binding energies of −20.5260 kcal/mol (Fig. S8A and B). Specifically, PA bound to the ARG606 residue of TLR4 through a pair of salt bridges, and LEU553 and PA had a pair of alkyl hydrophobic effects (Fig. S8A). These results imply that PA could directly bind to TLR4. Altogether, these results indicate that *AKK* improves microglial activation through inhibiting the TLR4/NF-κB inflammatory pathway by producing PA, which may contribute to the protective effects of PA on ARDS-related depression.

## Discussion

**Fig. 7.**
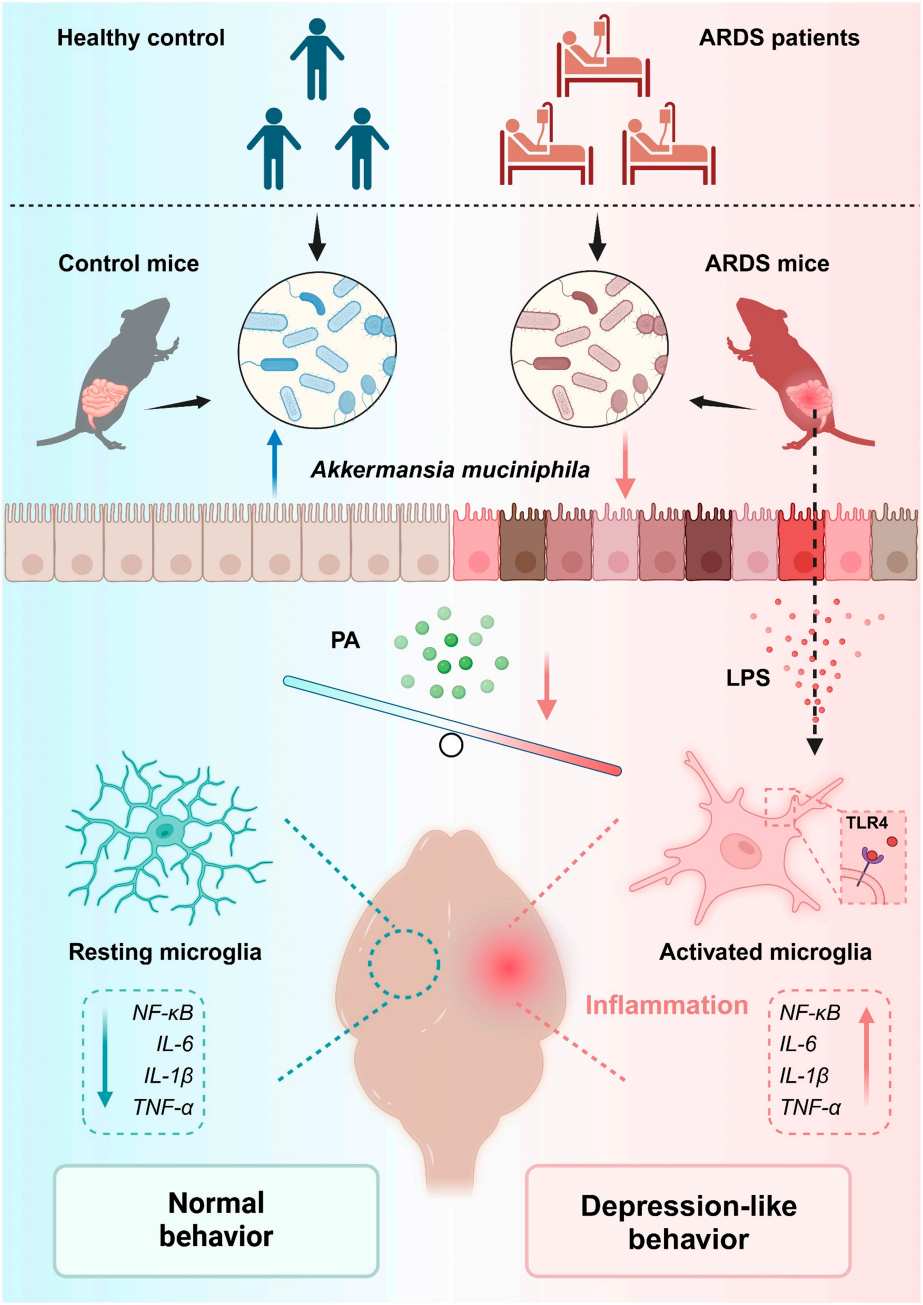
The microbiota–gut–brain axis regulates long-term depressive-like behaviors and neuroinflammation induced by ARDS, and supplementation of *AKK* and PA may be a promising therapeutic intervention strategy (image created with BioRender with permission).

In this study, we present compelling evidence showing that ARDS patients and animal models had altered gut microbiota and decreased levels of *AKK* and its metabolite PA. These alterations were associated with depression phenotypes. In addition, FMT from ARDS patients caused neuroinflammation and behavioral abnormalities with impaired intestinal barrier function in naive mice. Moreover, we observed that supplementation with the probiotic *AKK* could increase PA to ameliorate the inflammatory response of microglia and alleviate depressive-like behaviors. This study provides a potential therapeutic strategy for reducing the long-term psychiatric complications caused by ARDS and improving patient outcomes by supplementing *AKK* targeting the microbiota–gut–brain axis (Fig. 7).

There is growing evidence that ARDS survivors in ICUs are at increased risk for long-term psychopathology problems after discharge [3,31,32]. These psychiatric symptoms in turn delay the rehabilitation process. However, there are few reports about the mechanisms underlying ARDS-related mental illness. Dysbiosis of gut microbiota has been reported to be a common pathophysiological phenomenon in critically ill patients [33]. For instance, Zheng et al. [21] found that patients with ARDS caused by community-acquired pneumonia had gut microbiota dysbiosis characterized by the elevated abundance of Gram-negative bacteria, such as *Proteobacteria* and *Bacteroidota*. Additionally, Hu et al. [34] found that functional alterations in the gut microbiota of acute pancreatitis patients who developed ARDS were characterized by an increased abundance of *Proteobacteria*, *Enterobacteriaceae*, *Shigella*, and *Klebsiella pneumoniae*, and a decreased abundance of *Bifidobacterium* was important for ARDS-related depression symptoms. It is worth noting that varying changes in gut microbiota in ARDS were reported in different studies, probably due to the distinct underlying diseases causing ARDS.

On the other hand, the gut microbiota has also been implicated in many neurological disorders, such as autism, multiple sclerosis, Alzheimer disease, and neuropathic pain [35–37]. Brain health may be affected by the gut–brain axis in patients with ARDS [21,38]. At present, only a small number of studies have focused on the link between gut microbiota and neural damage in ARDS patients [21]. In this study, by transplanting the feces of ARDS patients into mice, we demonstrate an important role of gut microbiota for the pathogenesis of ARDS-related depression. We provided evidence that changes in ARDS lead to down-regulation of the diversity and relative abundance of several gut probiotics, including *AKK* (Fig 1). *AKK* is a well-recognized probiotic known for its anti-inflammatory properties and its ability to regulate digestion, immunity, and metabolism in the gut [39]. *AKK* has also been reported to improve cognitive dysfunction induced by high-fat diet-induced obesity and sleep deprivation in mice [40,41]. At the same time, probiotic supplementation is able to improve the anti-stress ability of brain tissue, relieve anxiety and depression, and improve intestinal microbial dysbiosis [42]. Whether probiotic supplementation can improve mental disorders caused by ARDS is currently unknown. In this study, *AKK* presents its potential to inhibit the development of long-term depressive-like behaviors in ARDS, suggesting that it could be a probiotic candidate for preventing psychiatric disorders induced by ARDS. Furthermore, *AKK* supplementation reduced LPS and inflammatory factors in mice with ARDS and activated microglia in their brains, and these findings suggest that *AKK* prevents ARDS-related neuroinflammation by reshaping the gut microbiota. Please note that, except *AKK*, other strains, including *Ligilactobacillus murinus* or *Muribaculum intestinale*, also showed down-regulation in the mouse model (Fig. 3B to D), whose potential influences on ARDS-related depression cannot be excluded at this stage, which warrants further investigations in the future study.

How exactly does *AKK* supplementation improve microglial activation? We used metabolomics to analyze the changes in gut metabolites in ARDS patients and mice. The results showed that PA levels in serum and fecal metabolites were significantly reduced in the depression group. Importantly, this phenomenon was reversed by *AKK* implantation. A previous study showed that the main metabolites of *AKK* were short-chain fatty acids (acetic acid and PA) under the dynamic culture of porcine mucin and human mucin [43,44]. PA is one of the typical short-chain fatty acids that is metabolized by many microorganisms. As reported, PA is mainly produced by *Bacteroidetes*, *Clostridium propionicum*, *Lactobacillus reuteri*, and *AKK* [27,45]. In the present study, we also found down-regulated *B. uniformis* in ARDS patients in addition to *AKK* (Fig. 1E). However, there was a positive correlation between *AKK* and PA, whereas no such correlation was observed with other microbiota (Fig. 1M). More importantly, the relative abundance of *B. uniformis* did not differ between ARDS patients with and without depression symptoms (Fig. S1A). These results suggest that *AKK*-derived PA plays an important role in ARDS-related depression. Nevertheless, we cannot exclude the possibility of other sources of PA that may also contribute to the process of ARDS-related depression. PA has been shown to easily cross the blood–brain barrier and affect brain function during development, health, and disease [46–48]. We found that PA pretreatment alleviated depressive-like behaviors and reduced microglia activation in ARDS mice. These results suggest that *AKK* preconditioning can reverse the decline in PA caused by ARDS, thereby alleviating neuroinflammation. Please note that *AKK* is reported to be involved in the production of various metabolites through tryptophan metabolism, short-chain fatty acid metabolism, and bile acid metabolism. Other metabolites except for PA were also changed in the ARDS patients and mouse model. Further investigations are warranted to dissect their potential effects in the development of ARDS-related depression symptoms.

Peripheral immune inflammation can aggravate neuroinflammation by promoting an active state of pro-inflammatory glial cells, which in turn can damage neural tissue [49]. The TLR4/NF-κB pathway is a critical component of the immune response, serving as a bridge between peripheral inflammation and central nervous system activities [50]. We found that microglia in ARDS mice showed an activated state (Fig. 2M). TLR4 is the receptor of LPS, which is expressed in microglia, astrocytes, and neurons [51]. Upon binding to the TLR4 receptor on microglia, LPS activates the downstream NF-κB signal transduction pathway, thereby releasing inflammatory factors IL-6, IL-1β, and TNF-α, which negatively affect emotional regulation. PA can enter the cell through the cell membrane through transporters and inhibit histone deacetylases to exert anti-inflammatory effects [52,53]. Hence, our study suggests a close relationship between PA and LPS molecules in inflammatory responses. Microbially derived PA, a pivotal signaling molecule in microbe–gut–brain communication, may target microglia to regulate brain inflammatory responses, influencing the emotional function of ARDS patients.

The mechanisms underlying depression induced by ARDS are complex. This study explores only partial functional characteristics of microglia in the cortex. Further investigation into other brain regions and related mechanisms is needed. Furthermore, due to the heterogeneity of ARDS patients and the limited number of clinical participants, coupled with the high variability of the human gut microbiome, there is a need for centralized research and long-term observation to explore changes in the gut microbiota of ARDS patients and the relationship between *AKK* and macrogenomic functions. Lastly, the potential contributions of the other microbiota and metabolites to ARDS related-depression as well as other related disorders warrant careful investigations in future studies.

In summary, we explored the underlying mechanisms of the effects of gut microbiota on depressive-like behaviors in ARDS mice. The specific combination of bacteria (*AKK*) and metabolites (PA) may serve as biomarkers for the onset of psychiatric disorders in ARDS patients, thus facilitating the development of precise prevention and treatment strategies.

## Methods

### Clinical sample collection

This study included ARDS patients admitted to the ICU of Southern Hospital, Southern Medical University, from November 2023 to March 2024. All patients met the Berlin diagnostic criteria for ARDS [54], including (a) acute onset < 1 week and (b) PaO_2_/FIO_2_ ≤ 300 mmHg and positive end-expiratory pressure ≥ 5 cm H_2_O. Exclusion criteria included (a) acute pulmonary edema due to heart failure, (b) sudden gastrointestinal discomfort or persistent diarrhea, and (c) receiving antibiotics or probiotic therapy for more than 1 week. This study focused on ARDS patients with secondary pulmonary infection in ICUs. We recruited age-matched healthy volunteers with no history of heart or respiratory diseases and normal lung function. Three months after ARDS survivors were discharged from the hospital, the patients were divided into a nondepression group (0 to 7 points) and a depression group (7 to 21 points) using the Hospital Anxiety and Depression Scale [55]. Samples of feces or anal swabs were collected on the admission day and stored at −80 °C until analysis on the following day. To obtain serum, blood samples were placed in a 5-ml vacutainer tube containing ethylenediaminetetraacetic acid (EDTA), mixed for 30 min in EDTA, centrifuged at 3,000 g for 10 min, and stored at −80 °C. Ethical approval for the study was obtained from the Ethics Committee of Nanfang Hospital, Southern Medical University (NFEC-202403-K2). All participants provided written informed consent for participation in the study. Detailed characteristics of recruited subjects are provided in Table S1.

### Mice management

C57BL/6 male mice of 6 weeks of age (18 to 22 g) were obtained from the Guangdong Medical Laboratory Animal Center (Guangzhou, China). All experimental procedures adhered to the Guide for the Care and Use of Laboratory Animals and received approval from the Institutional Animal Care and Use Committee of Southern Medical University (No. 2024-0112). Mouse feces were collected once a week from the anus once a week at a consistent time (1500 to 1600). The mice were euthanized after behavioral testing, and their lung tissues were washed with phosphate-buffered saline (PBS) through a tracheal tube, and bronchoalveolar lavage fluid was collected. Additionally, serum, lung, brain, and intestinal tissues were extracted and analyzed as described subsequently. In each group of mice, at least 9 independent experiments were carried out after establishing appropriate control groups.

Mice that underwent FMT were administered antibiotic-laced drinking water, containing ampicillin (1 g/l), vancomycin (500 mg/l), ciprofloxacin HCl (200 mg/l), and imipenem (250 mg/l) for a duration of 14 d to suppress their gut microbiota [56]. After 1 week of acclimatization, one group received fecal microbiota from ARDS patients, and the other group received fecal microbiota from the healthy controls (*n* = 10). Each mouse was given 200 μl of microbial suspension 3 times a week (on the 1st, 3rd, and 5th days) for 4 consecutive weeks. We chose a dose of 1 × 10^8^ colony-forming units (CFU)/mice based on previous reports [57–60]. For *AKK* administration, the *AKK* group was orally administered 200 μl of *AKK* (5 × 10^8^ CFU/ml), while the control group was orally administered 200 μl of PBS, 3 times a week for a total of 4 weeks. Mice received a daily oral dose of 100 mg/kg body weight of PA (Sigma-Aldrich, St. Louis, MO, USA) mixed with normal saline for a duration of 4 weeks (*n* = 9).

### Mouse model of ARDS

To simulate the pathophysiological processes of ARDS patients in the ICU, this study employed a “2-hit” mouse model to reproduce the immune status of patients (*n* = 10). As mentioned in previous studies [25], mice were randomly assigned to 2 groups and anesthetized with isoflurane. One received an intratracheal instillation of 0.1 N HCl (2 ml/kg), followed by a subsequent instillation of 1 mg/kg of LPS 24 h later. The other received an intratracheal instillation of saline only. Behavioral tests and tissue collection were performed 4 weeks after ARDS induction.

### Behavioral testing

#### Open field test

Mice were placed in the center of an open field arena (40 × 40 × 40 cm) and were allowed to explore their surroundings for 15 min freely. The path of movement was captured through an overhead video system (EthoVision XT 14.0), allowing for the analysis of the proportion of both distance and time spent in the central zone, as well as the overall distance covered. Following each test, the arena was cleaned with 75% ethanol to eliminate olfactory cues from the apparatus.

#### Elevated plus maze

The EPM used in this study comprises 4 arms arranged as 2 open and 2 enclosed sections. During testing, mice are introduced to the central platform and permitted to freely navigate the maze for 5 min, while their movements are tracked by the EthoVision XT software to quantify behavior. Animals falling from the exposed areas are omitted from the dataset. To prevent olfactory cues influencing subsequent trials, the apparatus is sanitized between uses.

#### Tail suspension test

Mice had their tails taped and then were hung on a rack at 30-cm height. A camera monitored them for 6 min; immobility time was recorded.

#### Forced swimming test

Mice were individually submerged for 6 min in a transparent water tank (12-cm diameter, 25-cm height, 15-cm water depth, 25 ± 1 °C). Immobility time in the last 4 min was recorded using the EthoVision XT software.

#### Sucrose preference test

Over a span of 2 d, individual housing ensured that mice had unlimited access to sustenance. For 24 h, they encountered 2 vessels—one holding 2% sucrose solution and the other, water. Afterward, the containers’ locations were altered. Liquid intake was monitored at daily intervals. Sucrose and H_2_O consumption data were collected and analyzed. Preference for sweetener (expressed in %) was computed by dividing sucrose ingestion by the total fluid volume consumed and then multiplying by 100%.

### Quantification of *AKK* in cecal content

After behavioral testing, mouse feces were collected for analysis. Genomic DNA was isolated from these contents using a stool DNA kit (D4015, Omega, China). The DNA templates were diluted to a concentration of 1 ng/μl with RNase-Free Distilled Water, and each sample was prepared in triplicate by loading 2 μl per well. The forward primer 5′-CAGCACGTGAAGGTGGGGAC-3′ and the reverse primer 5′-CCTTGCGGTTGGCTTCAGAT-3′ were premixed with SYBR Green Nucleic Acid Gel Stains (SYBR Green) Premix (AG11701, Accurate Biology, China). qPCR was conducted using a Bio-Rad CFX96 thermal cycler (Bio-Rad, Hercules, CA, USA) in a 20-μl reaction volume, with an initial step at 95 °C for 30 s, followed by 40 cycles of 95 °C for 5 s and 60 °C for 30 s. A melting curve analysis was performed to confirm the specificity of the amplification. To construct a standard curve, plasmid DNA containing the conserved sequence of *AKK* was prepared in a dilution series, enabling calculation of the absolute copy number of the 16S rDNA gene for each sample.

### *AKK* culture and oral supplementation

*AKK* cultures and oral supplements were previously described with some modifications [41]. The *AKK* strain (BAA-835, American Type Culture Collection, USA) was grown in brain heart infusion broth with 0.1% pig stomach coating added for better growth; it was kept without oxygen at 37 °C. Bacterial suspension for oral administration was prepared in PBS. A spectrophotometer (NanoDrop 2000c, Thermo, USA) was used to obtain a suspension with an optical density of 10^9^ CFU/ml at 600 nm.

### Cell culture and treatment

As previously described, brain microglial cells were isolated from P2 mice [61]. The cortices of newborn puppies were dissected and chopped into fine pieces using a dissection medium that included Hank’s balanced salt solution, 10% mM Hepes, and 1% (v/v) penicillin/streptomycin. After a 15-min incubation at 37 °C with 2.5% trypsin, a trypsin inhibitor (1 mg/ml) was introduced for 1 min. After centrifugation at 1,500 rpm for 5 min, the pellet was triturated and suspended in complete media (Dulbecco’s modified Eagle medium with 10% fetal bovine serum and 1% (v/v) penicillin/streptomycin). Subsequently, cells were plated onto 6-well plates that had been coated with poly-d-lysine at a density of 50,000 cells/cm^2^ to create mixed glial cultures. After a week of cultivation, microglia were extracted from the mixed glial cultures by agitating the confluent flask at a speed of 250 rpm for 2.5 h, performing this procedure twice with a 48-h gap in between. In the experiment, PA (117 μmol/l) (Sigma, Germany) was added to the cultured microglia cells for 15 min, followed by stimulation with LPS (100 ng/ml) or LPS + TLR4 inhibitor (10 μM; TAK-242) for 24 h [62]. Cells were fixed with paraformaldehyde and subjected to immunofluorescence staining. The supernatant was collected from the culture for enzyme-linked immunosorbent assay (ELISA) analysis, and western blot was performed to identify signaling pathway proteins, specifically TLR4, p-IκB, and p-P65, present in the cells.

### Histological analysis and immunofluorescence staining

Mice were anesthetized deeply with isoflurane (approximately 15 s), followed by rapid perfusion with physiological saline for 3 min and 4% paraformaldehyde solution for 4 min. Brain, colon, and lung tissues were removed, fixed again, and dehydrated in a 30% sucrose solution. Lung tissues were sectioned into 4-μm slices, while brain and intestinal tissues were sectioned into 15-μm slices. Lung tissue was stained with hematoxylin and eosin to observe changes in the extent of alveolar lesions. For microglia observation in brain tissue, sections were incubated overnight at 4 °C with anti-CD68 antibody (Servicebio, GB113109, 1:100) and anti-ionized calcium binding adaptor molecule 1 (anti-Iba-1) antibody (Servicebio, GB12105, 1:300). To assess the intestinal barrier integrity, colon sections were incubated overnight at 4 °C with primary antibodies ZO-1 (Servicebio, GB111402, 1:2,500) and occludin (Servicebio, GB111401, 1:2,500). Slides were immersed in PBS (pH 7.4) on a decolorizing shaker and subjected to three 5-min washes. Sections were then treated with Alexa Fluor 488-conjugated goat anti-rabbit immunoglobulin G (IgG) (H + L; 1:400) and incubated at 37 °C for 1 h. Following incubation, slides were washed with PBS and stained with 4′,6-diamidino-2-phenylindole (Southern Biotech, USA) staining solution for 10 min at room temperature without light.

### 16S rDNA sequencing analysis

DNA was extracted from samples of small intestinal contents. To create sequencing libraries, the 16S rRNA gene was amplified using universal primers, specifically 27F (5′-AGRGTTTGATYNTGGCTCAG-3′) and 1492R (5′-TASGGHTACCTTGTTASGACTT-3′). After preparing the library, a quality check was carried out prior to sequencing on a PacBio Sequel platform. Subsequently, we conducted a class-level analysis using the unweighted pair-group method with arithmetic mean and visualized the distribution of genera through bar graphs, all through the BMKCloud platform (www.biocloud.net).

### Nontargeted metabolomic profiling

The fecal matter or serum was thoroughly blended with a methanol solution that had a concentration of 12.5 μg/ml of 1,2-13C-myristic acid. Following centrifugation at 12,000 rpm for 15 min, the supernatant was retrieved for analysis via liquid chromatography–mass spectrometry (MS). The supernatant was examined using Agilent 6538 UHD and Accurate-Mass quadrupole time-of-flight/MS. Total ion chromatography spectra were gathered in both positive and negative ion modes. The initial data collected through MS underwent preprocessing with the MassLynx software. It was subsequently transferred to the Progenesis QI software (version 2.3) for processing, which encompassed tasks such as peak extraction, alignment, adjustment, and standardization. Metabolites were recognized through the analysis of retention time, precise mass, and tandem MS data, which were then compared to both the METLIN database and a custom-built database.

### Correlation analysis

Spearman’s correlations were computed and visualized in R (v4.2.0) to explore the relationships between the modified microbiota genera, altered metabolites, and indicators of depressive-like behaviors. A *P* value threshold of less than 0.05 was established to indicate statistical significance.

### Reverse transcription qPCR

Colon tissue samples were subjected to RNA extraction utilizing TRI Reagent (T9424, Sigma-Aldrich, China). This was followed by the conversion of RNA to complementary DNA through the HiScript II Q RT SuperMix for qPCR kit (Vazyme, China). Subsequently, qPCR was performed employing the ChamQ Universal SYBR qPCR Master Mix and analyzed on a qPCR detection system (Thermo Scientific NanoDrop 2000C). The relative expression of target genes was quantified via a standard method involving normalization with an internal control gene (β-actin) and calculation according to the 2^−ΔΔ*Ct*^ formula.

### Western blotting

Protein samples extracted from cortex tissue in radioimmunoprecipitation assay buffer, supplemented with a protease and phosphatase inhibitor cocktail, were subsequently quantified using a bicinchoninic acid protein assay kit. Equal quantities underwent sodium dodecyl sulfate polyacrylamide gel electrophoresis for 1 h at a low voltage followed by 2 h at a higher voltage before transfer onto cellulose sheets that were blocked with detergent-modified buffered saline + skim milk powder for an hour. These were probed with specific rabbit antibodies against proteins of interest (TLR4, 1:1,000, Abcam; p-IκB, 1:1,000; p-P65, 1:1,000; β-actin, 1:8,000, Abcam) at varying dilutions and then washed thrice and exposed to goat anti-rabbit/mouse secondary antibodies for another hour. Immunoreactive bands were visualized using chemiluminescence substrates. Densitometric analysis was performed using the ImageJ software.

### Enzyme-linked immunosorbent assay

The prefrontal cortex was extracted quickly, frozen instantly with liquid nitrogen, and then spun at a high speed. We measured protein levels and used ELISA kits (Jianglai, Shanghai, China) to quantify inflammatory markers like interleukins IL-6 and IL-1β, TNF-α, and LPS in these brain samples, following the kit’s directions. The *AKK* culture supernatant was centrifuged at 1,000×g for 20 min. The supernatant was then collected, and PA levels were quantified using an ELISA kit (RD-RX28534, Beijing RuiDaHengHui Science & Technology Development Co., LTD), following the manufacturer’s instructions.

### PA extraction and analysis

Mouse fecal samples (*n* = 5) were treated with ether and sulfuric acid. Following high-speed centrifugation, the ether layer was collected for PA concentration analysis using a gas chromatograph (Agilent model 6890 N, San Diego, CA, USA).

### Three-dimensional reconstruction

Microglia were imaged by confocal microscopy using a 0.5-μm step for optimal *z*-axis resolution. The Imaris 10.1.1 software (Bitplane) was used to analyze microglia morphology and 3-dimensional reconstruction [63,64]. Five mice were included in each group, and 2 sections from each mouse were randomly selected for imaging and quantification. To quantify the phagocytosis and activation status of microglia, we reconstructed the initially established thresholds for Iba-1+ microglia using the “Surface” function, which was rendered to a 3-dimensional surface, and reconstructed the CD68+ regions with the “SPOTS” function. The number of CD68+ spots within microglia was then measured using the “Split into Surface Objects” plug-in. We then used the “filament” function to render the cell body and quantify the microglial branch diameter, branch level, branch length, and number of branch points. Finally, the diameter and branch grade information were pseudo-color coded.

### Molecular docking

Molecular docking was used to simulate the interaction of PA binding with TLR4. The structure of PA was derived from the PubChem database. The TLR4 protein and structure were obtained from the Research Collaboratory for Structural Bioinformatics database (http://www.rcsb.org/). The Discovery Studio software was used to confirm the molecular docking of PA with TLR4. We set the criterion that docking energy values lower than 7.0 kcal/mol represent a strong binding interaction. Before docking, the ligands and protein (Protein Data Bank ID: 3FXI) were prepared using Discovery Studio 2021. Molecular docking was performed according to the standard default setting within 100 genetic algorithm runs of molecules in GOLD 5.1. A maximum of 125,000 operations was performed with annealing parameters as default for each genetic algorithm run. The cutoff values for hydrogen bonds and van der Waals interactions were set to 3.0 and 4.0 Å, respectively. When the top 10 solutions possessed root mean square deviation values within 1.5 Å, docking would be terminated. the ligand–protein complexes were mapped via the PyMOL 2.5 software.

### Statistical analysis

The data were analyzed using GraphPad Prism version 9 (GraphPad Software, La Jolla, CA, USA) and reported as mean ± standard error of the mean. The Student *t* test was used to assess significant differences between the 2 groups, and one-way analysis of variance was used to analyze multiple comparisons before Tukey’s post hoc multiple-comparisons test was applied. Analysis pertaining to the microbiota were conducted using Welch’s *t* test. Spearman’s correlation coefficient was then used to examine the relationships between blood metabolite intensities and genus levels. *P* values less than 0.05 were regarded as statistically significant.

## Data Availability

All data are present in the paper and the Supplementary Materials and can be available from the corresponding authors upon reasonable request.
