# Supplementary material for: Altered Gut Microbiota Contributes to Acute-Respiratory-Distress-Syndrome-Related Depression through Microglial Neuroinflammation
Source: Research (Wash D C). 2025 Mar 19;8:0636. doi: 10.34133/research.0636 (PMC11919824; doi:10.34133/research.0636)
Supplement: Supplementary 1 — Tables S1 and S2 Figs. S1 to S8 [file research.0636.f1.docx]

**Supplementary Material**

**Supplementary Table 1 Clinical characteristics of participants.**

**
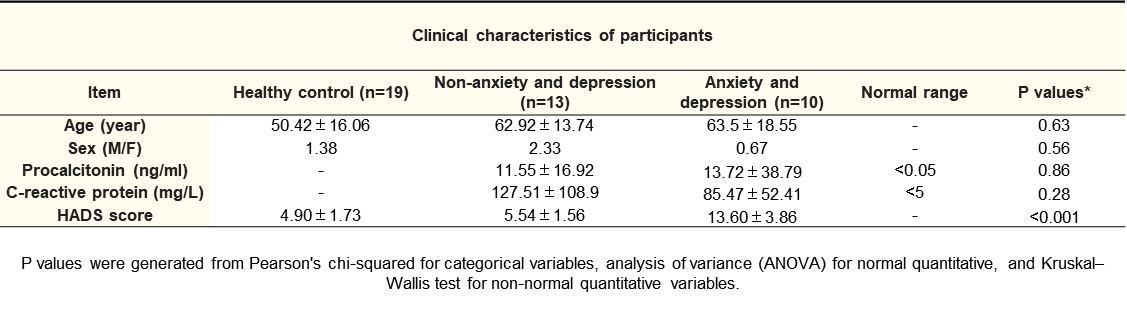
**

**Supplementary Table 2 Metabolites with consistent changes in the serum and feces of human samples**

**
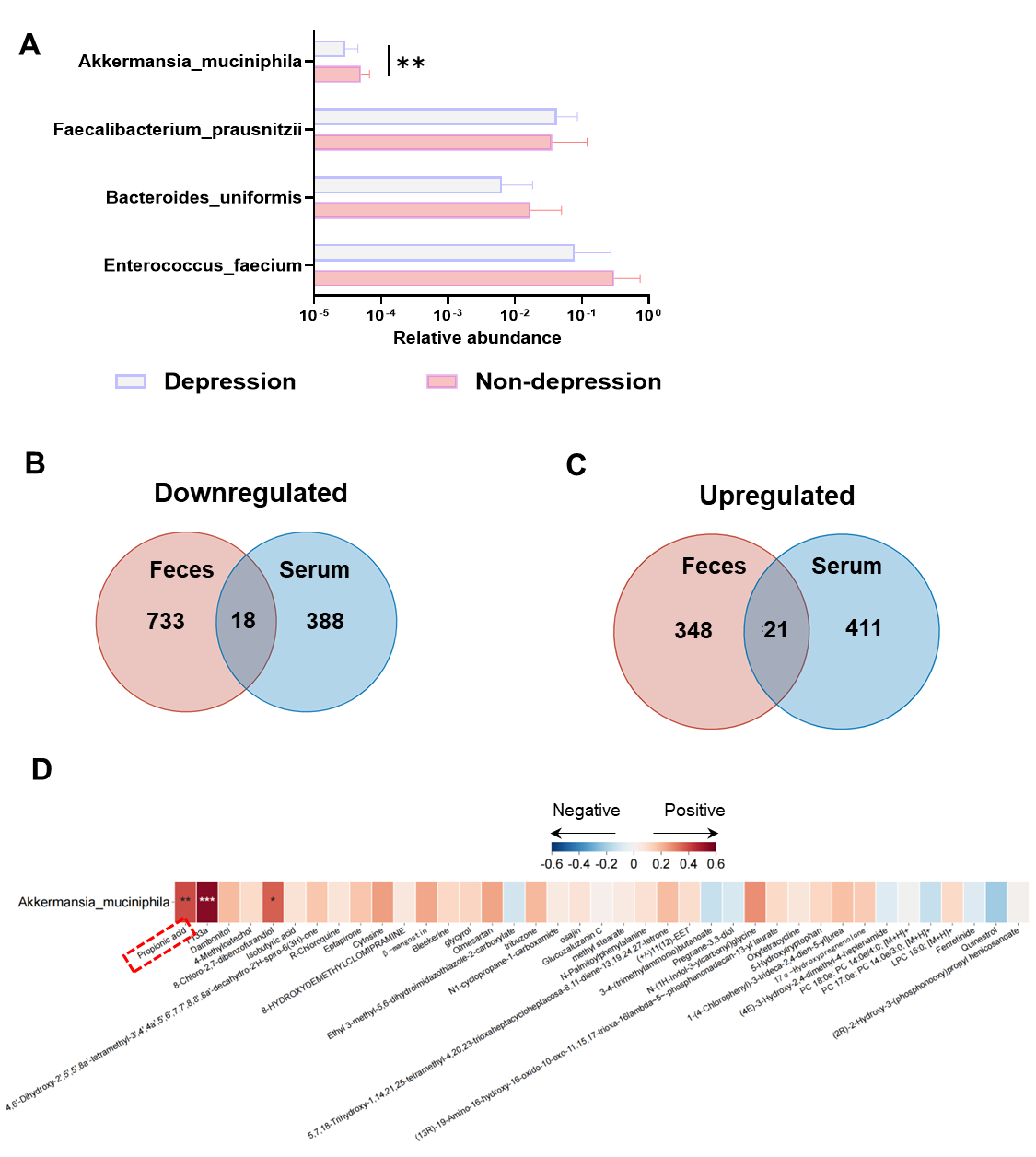
**

**Supplementary figure 1** (A) The relative abundance of gut microbiota at the species level in depressed and non-depressed groups. (B) Venn diagram indicates the intersection of downregulated metabolites in feces and serum. (C) Venn diagram indicates the intersection of upregulated metabolites in feces and serum. (D) Correlation of both upregulated and downregulated metabolites in feces and serum. **P < 0.01.

**
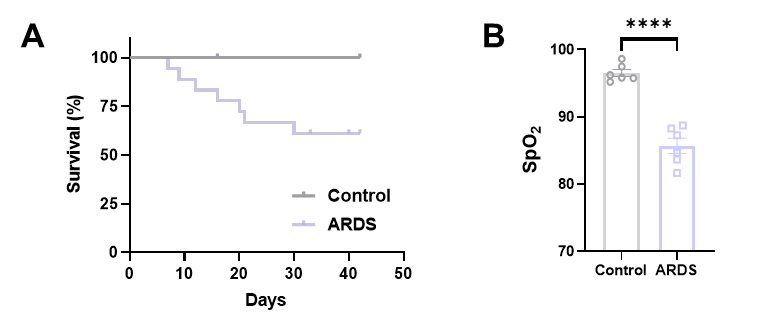
**

**Supplementary figure 2** (A) The survival rate of in the control and ARDS groups. (B) Pulse oxygen saturation (SpO2) measurements (n = 6). ****P < 0.001.

**
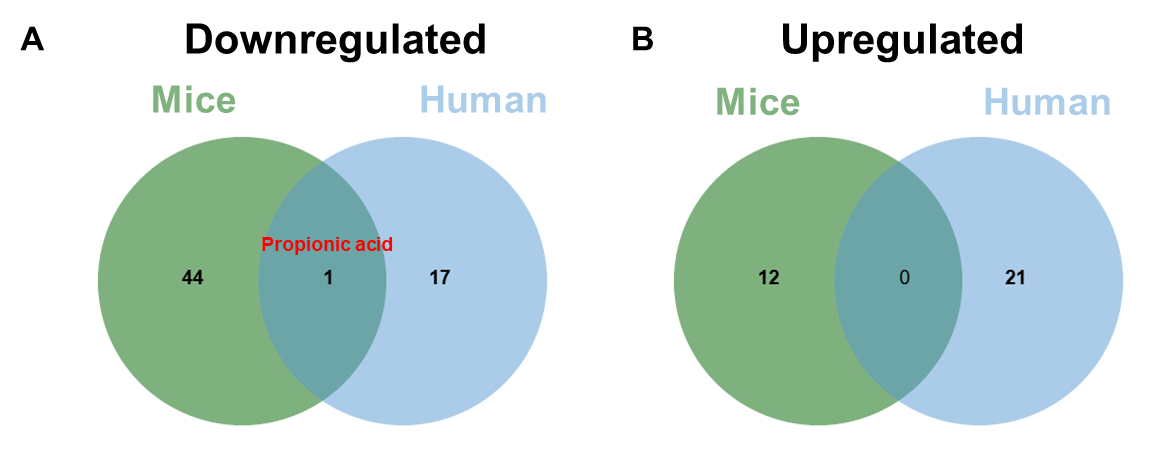
**

**Supplementary figure 3** (A) Venn diagram indicates the intersection of downregulated metabolites in human and mice. (B) Venn diagram indicates the intersection of upregulated metabolites in human and mice.

**Supplementary figure 4** PA levels in control group and *AKK* medium (n=6). ***P < 0.001.

**
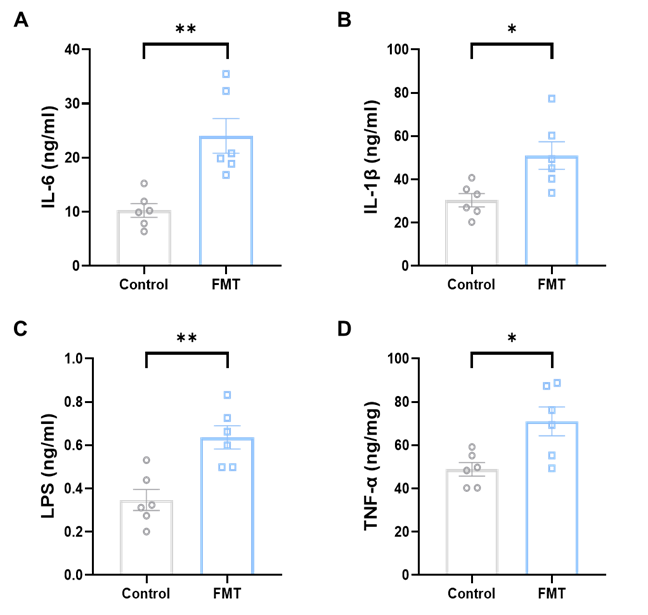
**

**Supplementary figure 5** (A-D) The levels of IL-6, IL-1β, LPS and TNF-α in FMT and control group (n = 6). *P < 0.05, **P < 0.01.


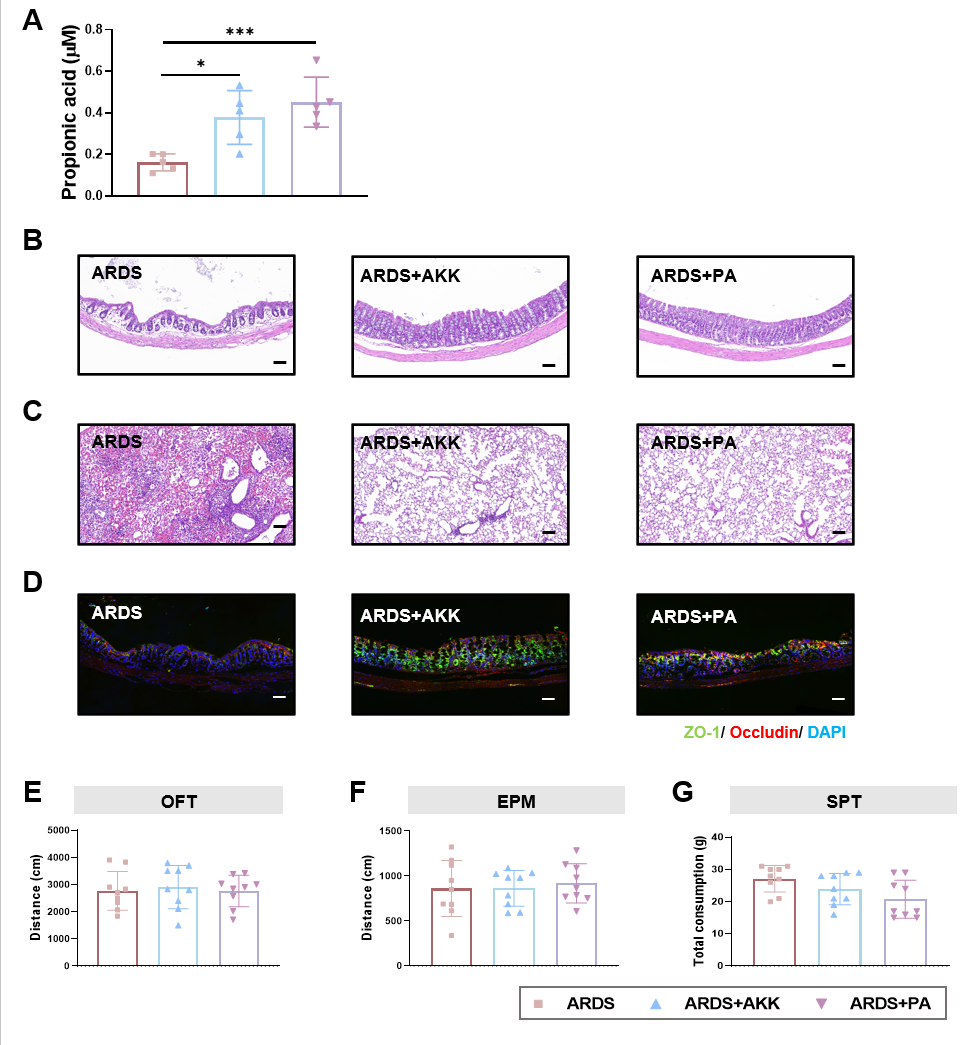


**S****upplementary figure 6** (A) Levels of PA in the serum of mice. (B) Hematoxylin-eosin staining was used to display the impairment of intestinal barrier. (C) Hematoxylin-eosin staining was used to display the severity of lung injury. (D) Immunofluorescence staining was used to detect the expression level of the tight junction protein ZO-1 (green) and Occludin (red) in the mice colonic tissues in each group. Scale bar = 100 μm. (E-F) Total distance of OFT and EPM. (F) Sucrose and total water consumption. (One-way ANOVA with Tukey’s multiple comparison tests, *P < 0.05, ***P < 0.001, ns means no significant).

.

**
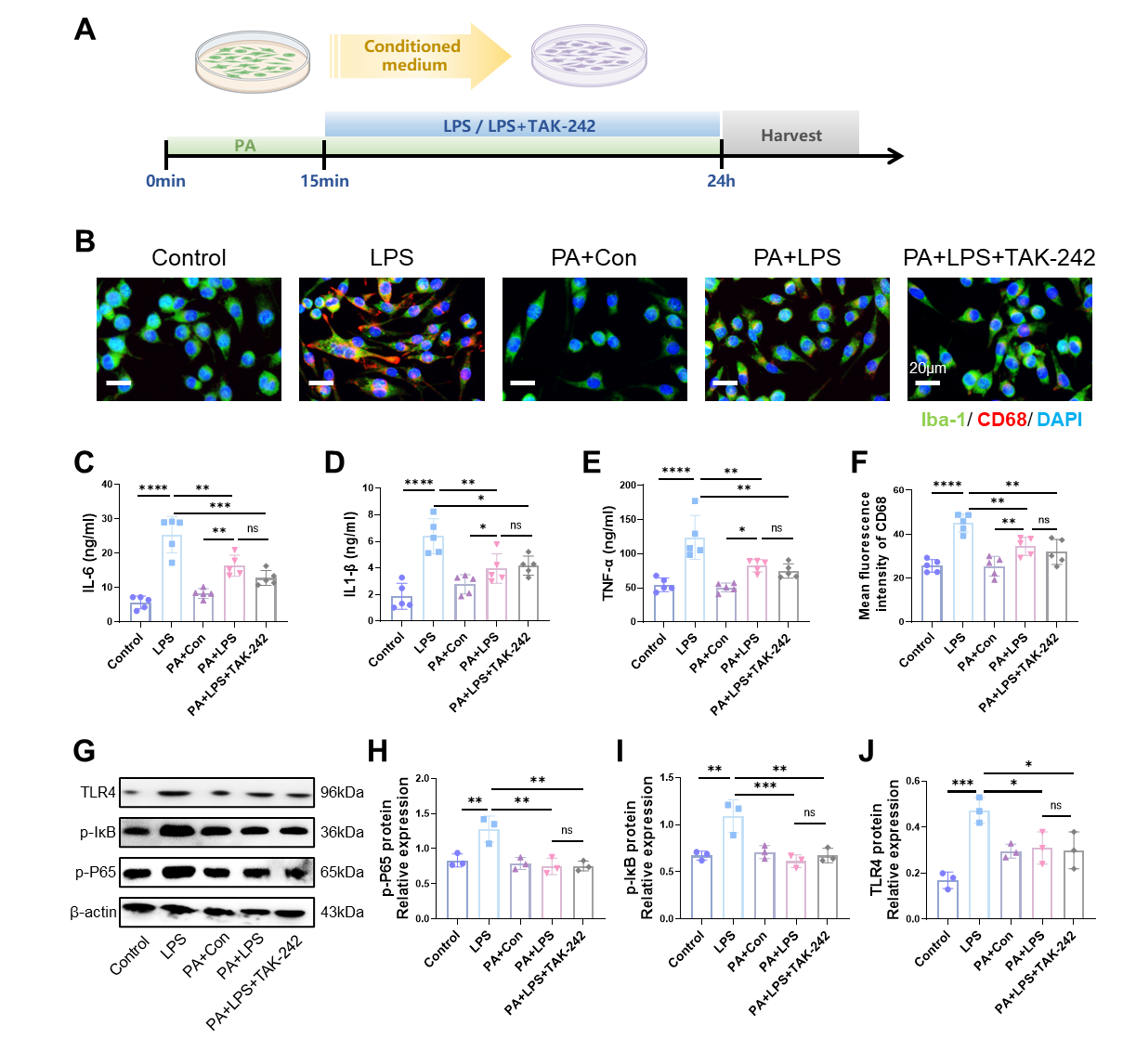
**

**Supplementary figure 7 *A. muciniphila* and PA improve neuroinflammation by inhibiting TLR4/NF-κB signaling in microglia.** (A) PA treatment of primary microglia. (B) Immunofluorescence micrographs of microglia (Iba-1, green; CD68, red). Scale bar = 20 μm. (C-F) Levels of IL-6, IL-1β, TNF-α and fluorescence intensity of CD68 in the microglia (n = 5). (G)-(H) Relative protein levels of TLR4, p-IκB and p-P65 in microglia by western blot (n = 3). (Student’s t test, *P < 0.05, **P < 0.01, ***P < 0.005, ****P < 0.001, ns means no significant).


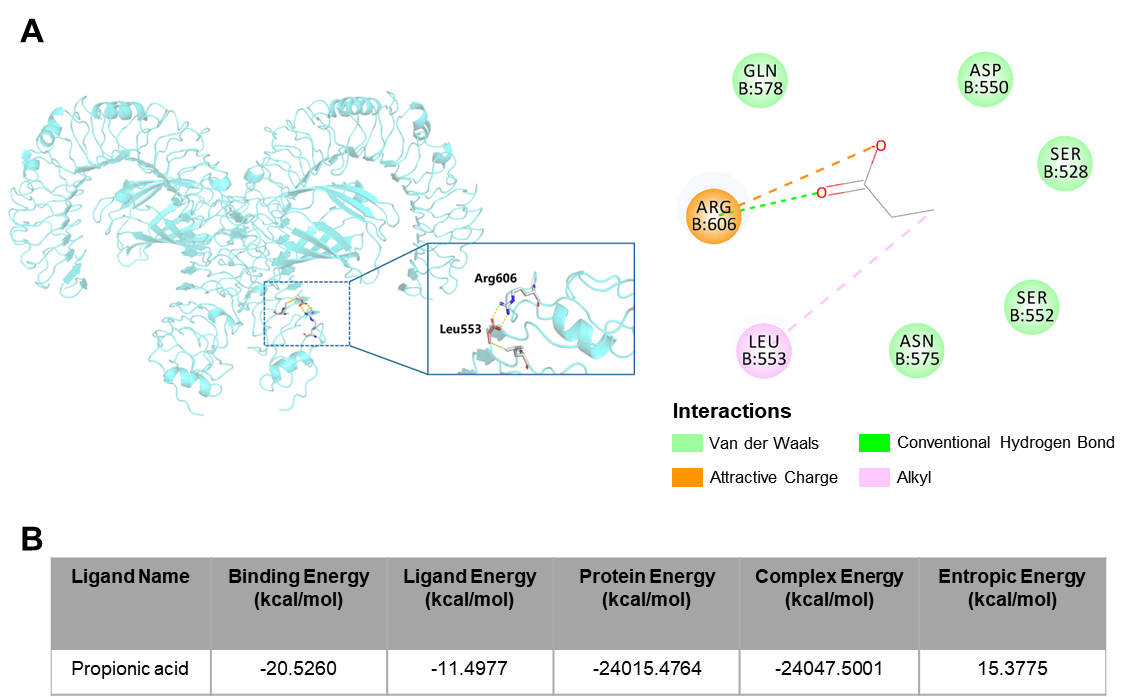


**Supplementary figure 8** (A) Molecular docking simulation of PA binding with TLR4 and binding diagram. (B) Molecular docking results of PA with TLR4.
